# Proposed RP–HPTLC–FLD kit for analysis of vitamin A palmitate in edible oils

**DOI:** 10.1007/s00216-025-05894-0

**Published:** 2025-05-19

**Authors:** Daniel Meyer, Sophia Hörnlein, Dietrich Rein, Gertrud E. Morlock

**Affiliations:** 1 Institute of Nutritional Science, Chair of Food Science, Justus Liebig University Giessen, Heinrich-Buff-Ring 26-32, 35392 Giessen, Germany; 2 BASF SE, Carl-Bosch-Str. 38, 67056 Ludwigshafen, Germany

**Keywords:** Food fortification, Vitamin A quantification, Retinyl palmitate, Edible oil, Sustainability, High-performance thin-layer chromatography

## Abstract

Preschool-aged children and non-pregnant women of reproductive age particularly in countries of a lower socio-demographic index may experience insufficient dietary vitamin A. Fortifying staple foods such as edible oils with vitamin A is a useful approach to reduce malnutrition. To enable better quality oil fortification, producers need reliable and inexpensive tools for assessment of the vitamin A contents of their products. A simple reversed-phase high-performance thin-layer chromatography fluorescence detection (RP–HPTLC–FLD) method was developed and studied for the quantification of vitamin A palmitate (VAP) in five edible plant oils. The challenging matrices of non-refined palm kernel oil and soybean oil, which are known to interfere with the accuracy of some easy-to-use test kits, were separated from VAP using the new RP–HPTLC–FLD method. The standard addition method provided good accuracy and acceptable calibration curves (coefficients of determination R^2^ between 0.9874 and 0.9999) even for manual application. The developed RP–HPTLC–FLD method proved simple, robust, sustainable, cost-efficient, and suitable for low-tech (minimum equipment) on-site analysis.

**Graphical Abstract:**

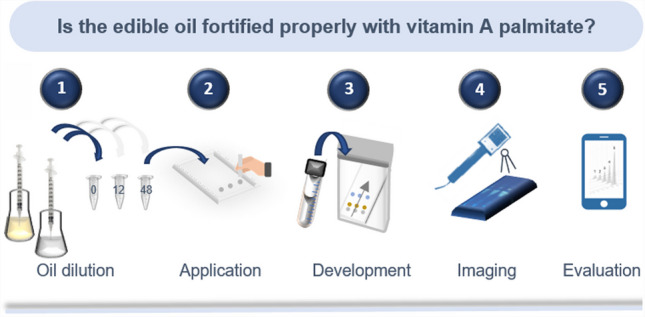

**Supplementary Information:**

The online version contains supplementary material available at 10.1007/s00216-025-05894-0.

## Introduction

Deficiency in vitamin A still threatens the health of preschool-aged children, non-pregnant women of reproductive age, and the general population, particularly in countries with a low socio-demographic index [1, 2]. Among five subcategories of nutritional deficiencies in children under 5 years, vitamin A deficiency accounted for the largest proportion of incident cases (62% in 2019) [3]. Large-scale food fortification can bring measurable improvements in vitamin A-dependent serum retinol concentrations and health status, particularly in children [4]. A sufficient supply of vitamin A is an investment in the future as it contributes to a healthier society. Vitamin A availability to those most in need has been a target for public and private stakeholders resulting in efforts including the fortification of staple foods [5]. Edible plant oil is a preferred vehicle for effectively supplying vitamin A to households of all incomes [6] based on its ubiquitous consumption and affordability [7]. Moreover, fortifying edible plant oil with the fat-soluble vitamin A was shown to be a technically feasible and cost-effective approach to supplying vitamin A [8]. It was adopted in voluntary and mandatory fortification efforts more than 20 years ago [5, 9]. For countries with a population at risk of vitamin A insufficiency, recommendations to produce vitamin A fortified edible oil range from about 30,000 to 100,000 IU/kg (9–30 mg RE/kg) [10, 11].

Efficient and safe fortification of cooking oil relies on the ability of producers and distributors to monitor the vitamin A content of their products at the site of manufacturing or repackaging [12]. Small and medium-sized oil mills and distributors often depend on inexpensive quantitative tests that are easy to use. However, most available quantitative analytical techniques require access to well-equipped laboratories for preparing and analyzing the samples. Efforts are ongoing to simplify the sophisticated, time-consuming, and expensive high-performance liquid chromatography fluorescence detection (HPLC–FLD) method to quantify stable and lipophilic vitamin A derivatives, *e.g*., vitamin A palmitate (VAP) [13]. Inexpensive and easy-to-use test systems for qualitative or (semi-)quantitative assessment of vitamin A or its derivatives in edible plant oils have been developed as follows. Photometric methods are low-cost but often need prior saponification and extraction thereby reducing the precision and method efficiency [14]. Field-ready portable testing devices can provide acceptable approximations of vitamin A contents in common oils used for food fortification when compared to HPLC [15]. The (semi-)quantitative point-of-need devices QuickView (Bagco, San Juan City, Philippines), and the Vitamin A Test Kit for edible plant oils (BASF, Ludwigshafen, Germany) are independent of instrumental laboratory equipment but require additional validation [16]. The iCheck (BioAnalyte, Teltow, Germany) was evaluated for analyzing VAP-enriched oils. However, details on the oil qualities used were not provided [17], complicating the assessment of matrix influences. For soybean oil, the accuracy was three times lower when compared with HPLC [17], likely due to known matrix effects [15]. Therefore, separating VAP from interfering components before analysis appears necessary. The methods mentioned above, which are based on the Carr–Price reaction of vitamin A or VAP with antimony trichloride or trichloroacetic acid [18, 19], work rapidly, are cost-efficient, and are suitable for most oils at low VAP contents of 3–30 mg retinol equivalents per kg (10,000–100,000 IU/kg) [15]. Unfavorable is the rapid fading of the formed color complex [18] and the suppression of the color signal by some oil matrices, as given for soybean oil [15]. Moreover, the reaction agents, including antimony trichloride, are less suitable for field testing due to their hazard categorization. Quantification of vitamin A at high contents is officially performed by photometric methods [20, 21] using the high absorption of the retinyl group at 326 nm in the UV spectrum. Unsaturated fatty acids in edible plant oils absorb UV light at only slightly lower wavelengths. With an increasing content of such fatty acids in the triacylglycerols, their UV absorption overlays the spectrum of vitamin A or VAP in fortified oils. Thus, a quantitative determination via spectrophotometry becomes difficult without prior separation of vitamin A or VAP from the interfering matrix.

Since the analytical systems are either expensive or not suitable for the quantification in all relevant plant oils, use hazardous chemicals, or require specialized and well-trained personnel, further techniques were explored. The separation of vitamin A or its derivatives from the interfering matrices of different edible plant oils by high-performance thin-layer chromatography (HPTLC) can serve as an efficient and affordable alternative to HPLC methods [22]. Therefore, a quantitative HPTLC method was targeted for the analysis of VAP in fortified edible plant oils. Different detection modes of vitamin A were studied, *e.g*., at its maximal UV absorption or via selective derivatization reactions. The method to be developed was intended to be cost-efficient, sustainable and suited for portable analysis with minimal risk of hazard.

## Materials and methods

### Chemicals and materials

Double-distilled water was produced by a Heraeus Destamat Bi-18e (Fisher Scientific, Schwerte, Germany). Vitamin A palmitate (VAP, ≥ 99.5%) was bought from Merck (Darmstadt, Germany). Acetone (≥ 99.5%), *iso*-propanol (≥ 99%), and n-heptane (≥ 99%) were purchased from Honeywell (Seelze, Germany). Ethanol (≥ 99.8%) was delivered from Sigma Aldrich (Darmstadt, Germany). HPTLC plates silica gel 60 RP-18 (briefly, HPTLC plates RP-18, 20 cm × 10 cm), and dichloromethane (≥ 99%) were obtained from Merck (Darmstadt, Germany). Lutein (L, analytical standard) was bought from Overseal Foods (Overseal, UK). α-Tocopherol (T, analytical standard) and β-carotene (C, analytical standard) were purchased from Merck & Cie (Schaffhausen, Switzerland). Acetic acid (99–100%) was bought from VWR International (Fontenay-sous-Bois, France). Edible plant oils from linseed (LO; Alnatura, 64,295 Darmstadt, Germany), sunflower seed (SO; Schwarzwaldmilch Offenburg, 77,652 Offenburg, Germany), refined/non-refined palm kernel oil (RPO; Landkrone Naturkost und Naturwaren, 66,386 St. Ingbert, Germany, and NRPO; KTC Edibles, Wednesbury, West Midlands, WS10 7DE, UK) and soybean (SOY; Henry Lamotte Oils, 28,197 Bremen, Germany) were tested.

### Fortification of oil samples with VAP

To obtain the fortified oil samples with the targeted VAP content of 25,000 International Unit (IU)/kg (13.75 mg/kg) and 65,000 IU/kg (35.75 mg/kg), a calculated volume (2.1–6.0 µL; Table S1, Supplementary Information) of the 1-mg/mL VAP standard solution was added to each measured weight from a 200-µL oil sample (154–184 mg, differences in densities due to different fat compositions of the oils and impurities especially in the unrefined palm oil). In the case of manual application, five additional samples were analogously prepared (Table S1, Supplementary Information) to contain additional VAP of 3, 6, 12, 24, and 48 ng/band when 2 µL of the fortified diluted oil sample was applied.

### Standard solutions

The four reference standards (VAP, L, T, and C) were dissolved each in *n*-heptane (1 mg/mL). The VAP standard was also prepared in *iso*-propanol (1 mg/mL) and diluted in the same solvent 1:10 (*V/V*) to obtain a 100 ng/µL solution. The VAP standard solution was diluted 1:100 (*V/V*) in *n*-heptane to obtain a 10 ng/µL solution. For high-resolution mass spectrometry, LO was diluted 1:100 (*V/V*) with *iso*-propanol.

### Demonstration of VAP fluorescence in oils

Each oil sample was diluted 1:10 with *n-*heptane (200 µL oil to 1800 µL *n-*heptane). The standard solutions (L, T and C 2.5 µL/band each; VAP 0.1 and 2.5 µL/band) and two times each diluted oil sample (1 µL/band each of diluted refined and non-refined palm kernel oil, linseed oil, sunflower seed oil and soybean oil) were applied on the HPTLC plate (Automatic TLC Sampler 4, CAMAG, Muttenz, Switzerland) and documented at FLD 366 nm (TLC Visualizer 2, CAMAG). Each second oil sample application was oversprayed and thus fortified with 0.3 µL VAP (0.3 µg/band each). The HPTLC plate was developed either with dichloromethane–acetic acid–acetone 2:4:5 (*V/V/V*) [23] or with ethanol up to 80 mm (Twin Trough Chamber, CAMAG) and documented at FLD 366 nm (TLC Visualizer 2, CAMAG).

### RP–HPTLC–FLD method

The diluted oil sample (2 µL/band) and standard solutions (0.3–2.5 µL/band) were applied on the HPTLC plate as 8-mm bands (Automatic TLC Sampler 4, CAMAG) or manually as spots (calibrated 2-µL pipette, Eppendorf, Hamburg, Germany). The plate was developed up to 80 mm (from the plate bottom, Twin Trough Chamber, CAMAG) using either ethanol (taking ca. 50 min) or a mixture of acetone–water 18:1 (*V/V,* taking ca. 15 min) and documented at FLD 366 nm (TLC Visualizer 2, CAMAG). Videodensitometrical profiles and calibration curves (peak areas) were obtained via visionCATS software (version 3.0, CAMAG). The x-axis intercept of each visionCATS calibration curve was calculated online at www.wolframalpha.com. To use the online resource, the internet address was typed into the internet browser (Firefox Browser Version 103.0, Mozilla Foundation, Mountain View, CA, USA), whereby the browser cache was deleted directly before the site was visited. The polynomial equation given by visionCATS was written in the data text field that appeared in the upper tenth of the display. The equation was written in the form of y = ax^2^ + bx + c. By clicking on the orange button, that can be found on the same text field, the x-axis intercept (y = 0) was calculated and called Root by the internet resource.

### High-resolution mass spectrometric evaluation

To further investigate the content of the HPTLC bands, high-resolution mass spectrometric (HRMS) analysis was performed of discrete HPTLC bands. The linseed oil (7 µL/band of 1:100 dilution in *iso-*propanol, *V/V*) and VAP (14 µL/band of 100 ng/µL in *iso-*propanol, *V/V*) as well as their mixture (*V/V*; 21 µL/band of linseed oil mixed 1:2 with VAP) were applied on the HPTLC plate (Automatic TLC Sampler 4). The plate was then developed with dichloromethane–acetic acid–acetone 2:4:5 (*V/V/V*) [23] up to 80 mm (Twin Trough Chamber) and documented at FLD 366 nm (TLC Visualizer 2). Bands of interest were eluted from the HPTLC chromatogram and online transferred to heated electrospray ionization high-resolution mass spectrometry (HESI-HRMS, QExactive Plus mass spectrometer, Thermo Fisher Scientific, Dreieich, Germany). Briefly, the respective zone of interest was eluted (200 µL/s, methanol) into the HESI-HRMS system via the PlateExpress interface (Advion, Ithaca, NY, USA). The HRMS instrument probe was set to + 3.5 kV and − 3.2 kV, 270 °C capillary and 200 °C probe heater temperature, resolution 280 000, m/z 100–1500, and automatic maximum injection time 10/200 ms for positive/negative ionization.

## Results and discussion

### Evaluation of Carr–Price-based tests and needs for improvement

A major problem during the quantification of vitamin A derivatives via the Carr-Price reaction is the fading of the blue color complex after 5–10 s. The reason for the instability of the retinyl complex is not known in detail. Moreover, the derivatization reagent may react with other substances present in the test matrix. Edible oils, including unrefined palm kernel oil and soybean oil, fail to produce a visible color complex with Carr–Price-type reagents, possibly due to oil components interfering with the reaction. To reduce the dependence of the Carr–Price reaction on the tested oils with their different components, a simple separation of the VAP from the potentially interfering matrix components of the oil followed by selective detection was targeted. The method to be developed should be inexpensive, sustainable, minimally hazardous, and suitable to develop and propose a test kit for portable analysis.

### RP–HPTLC–FLD method development

**Fig. 1:**
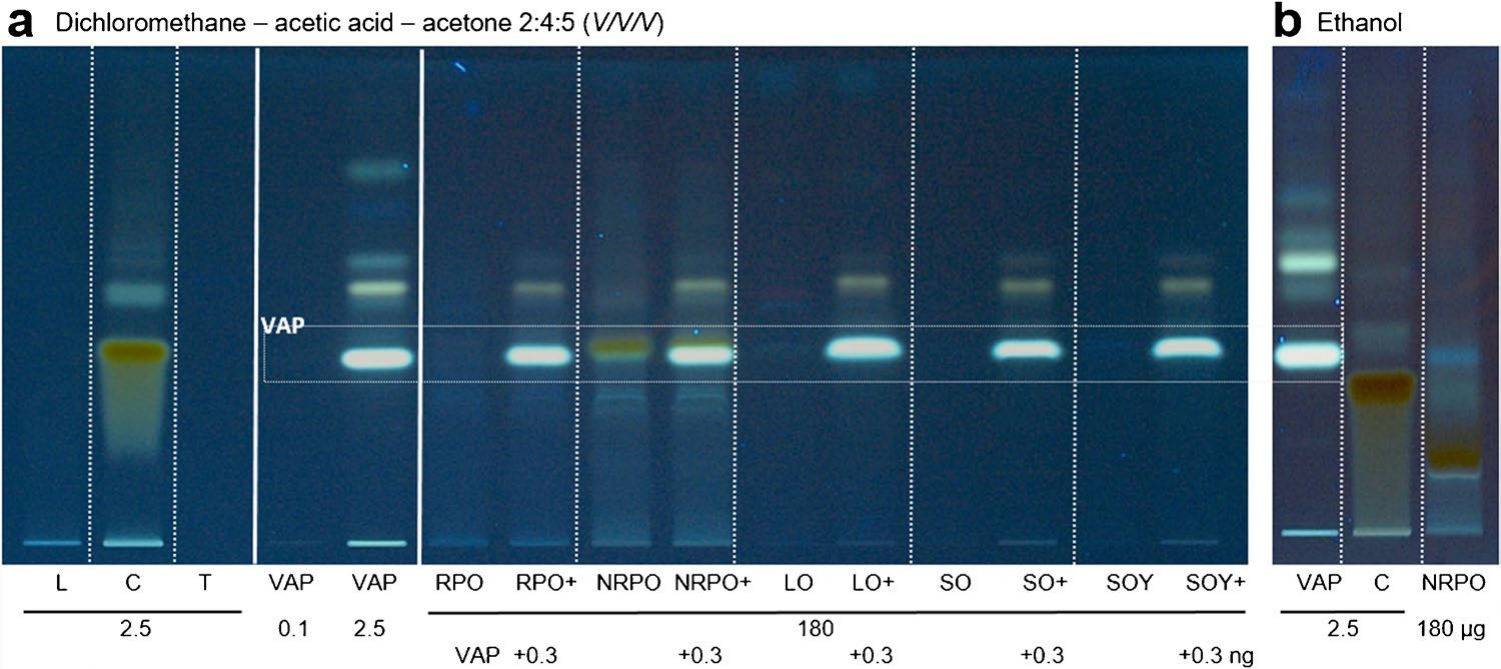
Demonstration of the VAP fluorescence in different plant oils: HPTLC chromatograms at FLD 366 nm on HPTLC plates RP-18 developed with **a** dichloromethane–acetic acid–acetone 2:4:5 (*V/V/V*) or **b** ethanol showing lutein (L), β-carotene (C), α-tocopherol (T), vitamin A palmitate (VAP), diluted refined palm kernel oil (RPO), non-refined palm kernel oil (NRPO), linseed oil (LO), sunflower seed oil (SO), soybean oil (SOY), and respective oils fortified with 0.3 µg VAP (+)

Different stationary and mobile phases as well as derivatization reagents were selected to find out how selectively the VAP can be separated and detected in the different VAP-fortified diluted oil samples (Figs. S1–S5, Supplementary Information). Different solvent systems from literature were studied on the reversed and normal phase as well as five different derivatization reagents, *i.e*., the iron (III) chloride/ferrozine reagent [24], the 4-methoxybenzaldehyde reagent [25], the antimony(III)chloride Carr-Price reagent [18, 19], the phosphomolybdic acid reagent [26] and the copper(II) sulfate [25]. They all point to double-bound systems of vitamin A and its derivatives and can therefore detect the vitamin and chemicals that have comparable molecular structures. In each HPTLC–Vis chromatogram after the respective derivatization, the VAP was revealed in the same color as other components of the VAP-fortified diluted oil samples, indicating matrix interference. This highlighted why separation is crucial since such derivatization tests performed in the liquid phase would lead to more or less oil-dependent inaccurate results. The VAP standard track showed several bands (in particular evident in Figs. 1 and S4, Supplementary Information), which was explained by the presence of different VAP isomers or VAP oxidation products. It was unclear whether these were already present in the standard or were formed during chromatography. The method’s effect on VAP would equally influence all samples and standards within the same oil matrix analyzed on the same plate. Since it is a relative evaluation and the respective lipophilic natural dyes and unsaponifiable matter remain constant, these factors do not impact the result. Hence, the main band of the VAP was utilized for subsequent quantification. Exemplarily, the highly absorbing minor components L, C, and T often present in oils (Fig. S4, Supplementary Information) were analyzed in comparison to the VAP but unfortunately showed the same hue as the VAP after derivatization with the iron (III) chloride/ferrozine reagent, which was not helpful for its selective detection. Among the studied mobile phases (Figs. 1a and S1–S4, Supplementary Information), ethanol was found to be acceptable as a simple, non-hazardous mobile phase (Figs. 1b and S4, Supplementary Information). However, it took 50 min up to 80 mm migration distance, which still needed further optimization as discussed later.

### Demonstration of VAP fluorescence in oils

Selective derivatization and normal phase separation were not pursued further, as it turned out that VAP fluoresced blue-green even without the slightest derivatization on the reversed-phase plate (HPTLC plate RP-18; Figs. S4 and S5, Supplementary Information). This VAP fluorescence observed in the different oils was, first, stronger on the reversed phase than on the normal phase (Figs. S3 vs. S4, Supplementary Information) and, second, stronger with oil matrix than without (Fig. S5, Supplementary Information). In a nonpolar surrounding, the VAP fluorescence was induced. Later, it was noticed that a recently published RP–HPLC–FLD method requiring a fully equipped HPLC apparatus [13] exploited a comparable phenomenon already described in 1998 [27], which, however, was not mentioned in the publication [13]. If VAP is dissolved in an organic solvent in the presence of ferrozine-Fe(II) complex and excited by UV light (excitation wavelengths and fluorescence depend on the solvent used) fluorescence can be recorded allowing for VAP quantification by external calibration [13, 24]. Finally, this VAP fluorescence, which is induced in a nonpolar surrounding (HPTLC plate RP-18 surface and oil as solvent), was found to be advantageous in saving time, chemicals, waste, and costs, and reduced health hazards, which have been requirements for the new test kit to be developed. The costs were further reduced by using a freeware software for subsequent quantitative analysis.

To demonstrate the apolar-induced VAP fluorescence, different diluted plant oil samples were applied as bands twice on the plate, whereby each second band was oversprayed and thus fortified with VAP (0.3 µg/band). Additionally, L, C, T, and VAP standard solutions were applied on the same HPTLC plate, which was then developed with dichloromethane–acetic acid–acetone 2:4:5 [23] (*V/V/V*) and detected at FLD 366 nm. The VAP showed a bright blue-green fluorescence (Fig. 1a). It was evident that VAP needed to be present at an amount above 0.1 µg/band to be detectable, but an amount of 2.5 µg/band showed already a too strong fluorescence. The non-fortified oils did not show any fluorescence at the *hR*_F_ value of the VAP standard band, whereas all oversprayed and thus fortified oils (with the 0.3-µg VAP) showed a strong fluorescence, which was almost as strong as the 2.5-µg VAP standard band. This enhancement of the VAP signal by the oil matrix was clearly observed in all five diluted oil samples. Fortunately, no further oil constituents interfered with the fluorescence detection of VAP. Only for the non-refined palm kernel oil, the β-carotene zone co-eluted with the VAP zone, which however, was resolved using the comparatively less toxic ethanol as mobile phase (Fig. 1b). Here, the β-carotene zone was separated and eluted below the VAP zone.

Linseed oil is not typically used for VAP fortification, but it is a challenging matrix due to its high content of lipophilic natural dyes and susceptibility to oxidation. In contrast, sunflower seed oil was expected to be less challenging and soybean oil is known to cause problems with Carr-Price-based test systems [15, 19]. Especially for soybean oil, it was crucial to separate the VAP from matrix interferences to enable quantitative analysis. For all these oils (Fig. 1), no interferences were observed using selective and sensitive fluorescence detection.

### Quantification via standard addition method

**Fig. 2:**
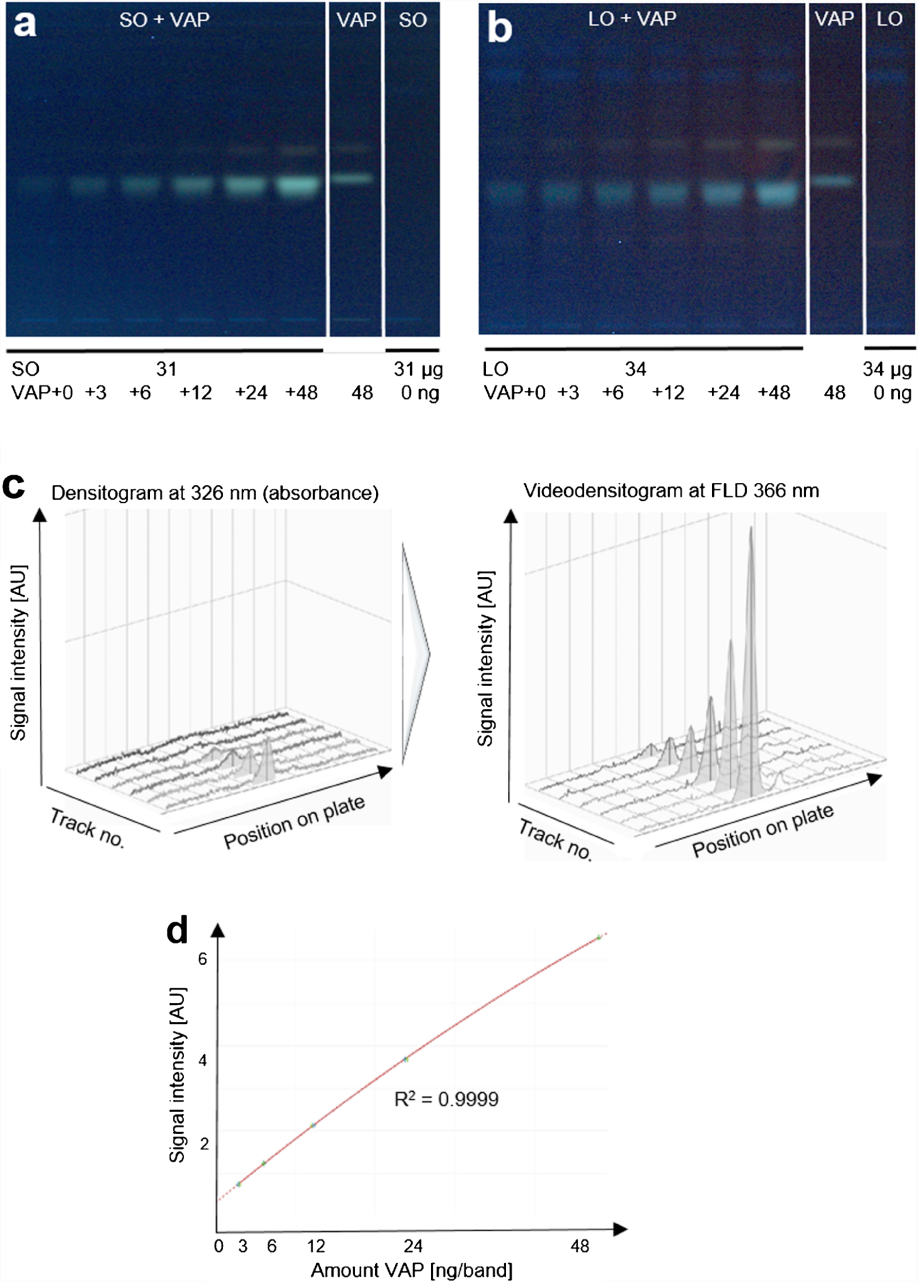
Response study for sunflower seed oil: **a** HPTLC chromatograms at FLD 366 nm of diluted sunflower seed oil (SO; 31 µg/band, 2 µL/band each) and linseed oil (LO; 34 µg/band, 2 µL/band each) spiked by overspraying with increasing amounts of vitamin A palmitate (VAP, 3–48 ng/band, 0.3–4.8 µL/band of a 10 ng/µL VAP in *n*-heptane solution) in comparison to the references VAP (48 ng/band) and the respective diluted oils on HPTLC plate RP-18 developed with ethanol, and **b** densitograms via absorption measurement at 326 nm, and **c** videodensitograms of peak profiles extracted from the image at FLD 366 nm and **d** corresponding calibration curve

As the apolar-induced VAP fluorescence was dependent on the oil matrix, a standard addition method was used for the accurate quantification of the VAP in the respective oil to compensate for the oil-dependent matrix influence on the fluorescence signal. For sunflower (Fig. 2a) and linseed seed oils (Fig. 2b), any interfering signals at the *hR*_F_ value of VAP were not detectable for the oil volume used (2 µL of the 1:10 diluted oil). Thus, no interferences were expected but need to be tested using exemplarily the sunflower seed oil for verification of the standard addition method. The sunflower seed oil (blank oil sample) was fortified at 25,000 IU/kg (2 µg VAP added to the measured weight of 200 µL oil) and diluted 1:10 with *n-*heptane. The fortified and diluted oil sample was divided into six 0.2-mL aliquots. One aliquot was left unspiked and the other five were spiked with five different VAP amounts to obtain the five different calibration levels. Alternatively, spiking by overspraying was tested. The fortified and diluted sunflower seed oil sample was applied six times (2 µL/band each; equal to 2 ng VAP per measured weight of 0.2 µL non-diluted oil), and thereof five bands were oversprayed with a different VAP volume to obtain the five different VAP standard addition levels of 3, 6, 12, 24, and 48 ng per measured weight of 0.2 µL non-diluted oil (Table S1, Supplementary Information). As a reference, the VAP and the diluted oil (not fortified and not spiked) were also applied. The plate was then developed with ethanol and the resulting HPTLC chromatogram was inspected at FLD 366 nm.

The following (video)densitometric evaluation of the HPTLC chromatogram provided increasing VAP fluorescence signal intensities for increasing VAP amounts in the fortified, diluted, and differently spiked sunflower seed oil. The densitograms obtained by absorption measurement at 326 nm (Fig. 2c), which is the absorption maximum of VAP [20, 21], were compared to the videodensitograms obtained from fluorescence signals (peak profiles extracted via videodensitometry from the chromatogram image at FLD 366 nm). As expected, the videodensitograms (fluorescence) showed comparatively more intense responses and a higher signal increase. The resulting polynomial calibration curve via the peak areas (Fig. 2d) had a coefficient of determination R^2^ of 0.9999. The intercept of the polynomial calibration curve with the x-axis (y = 0) was used to calculate the VAP content of the fortified oil. The x-axis intercept was calculated to be 2 ng/band. It accurately corresponded to the 2-ng VAP amount, with which the oil was fortified (Table S1, Supplementary Information). This proved that the quantification was accurate and verified the use of the standard addition method. Thus, the suitability of the method to quantify 25,000 IU of VAP per kg sunflower oil was successfully demonstrated. The standard addition method, for which all calibration levels were analyzed in the same apolar matrix (calibration in the matrix), was highly advantageous because it provided the accurate VAP value of the oil sample because the fluorescence enhancement was the same for the calibration standards as for the sample.

### Matrix influence and simplification of workflow steps

**Fig. 3:**
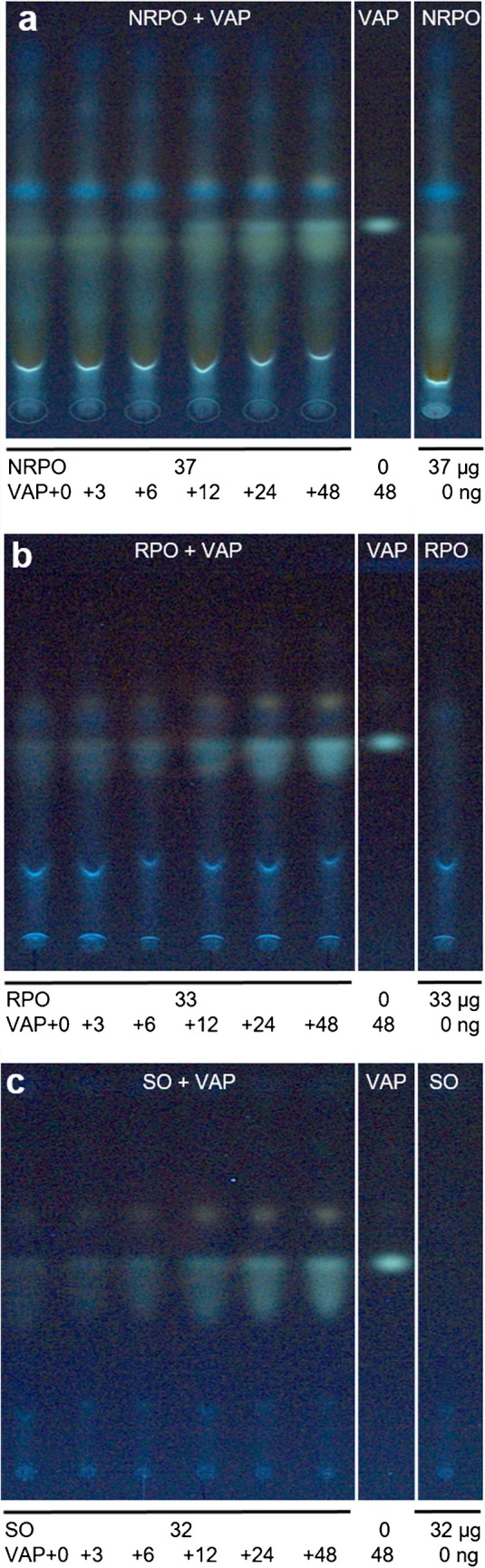
Manual application of samples: HPTLC chromatograms at FLD 366 nm on HPTLC plates RP-18 developed with ethanol showing **a** non-refined palm kernel oil (NRPO), **b** refined palm kernel oil (RPO), and **c** sunflower seed oil (SO), each diluted and spiked with increasing amounts of vitamin A palmitate (VAP) in reference to VAP and the respective diluted oil

The tested sunflower seed oil (Fig. 2a) showed no matrix effects that were detectable in the HPTLC chromatogram at FLD 366 nm. However, the β-carotene in the non-refined palm kernel oil was located just below the VAP zone (Fig. 3a). In contrast and as expected, the refined palm kernel oil showed fewer matrix effects and was quantitatively analyzable despite the tailing VAP zone (Fig. 3b), most likely caused by the manual application via a simple calibrated glass pipette (*e.g*., local plate overloading), if compared to the automated spray-on application (Figs. 1 and 2). The visible VAP tailing increased with increasing amounts of VAP added (Fig. 3). The VAP zone tailing had to be included in the peak area integration, as the presence of VAP was confirmed by high-resolution mass spectrometry using exemplarily a diluted linseed oil sample oversprayed with VAP at 1.4 µg/band. Interestingly, also oxidized VAP was found in the zone tailing (Fig. S5, Supplementary Information). The source of the oxidized VAP is unknown. For a relative evaluation (all on the same plate using the standard addition method), the oxidation was assumed to be without any influence on the results as long as the correlation coefficient is acceptable, as demonstrated later.

In the case of manual application, the glass capillaries used should be calibrated capillaries for discharge so that the volume applied to the HPTLC plate is correct. The solutions should be applied spot-wise at the same volume to generate comparable start zone geometries for all samples, which is recommended for good quantification. Therefore, the non-refined and refined palm kernel oil samples were fortified at 65,000 IU/kg and diluted (Table S1, Supplementary Information). To determine the fortified VAP amount via the standard addition method as a reference, five fortified sample aliquots each were spiked, which contained 3, 6, 12, 24, and 48 ng added VAP when 2 µL of the spiked fortified diluted oil sample was applied (Table S1, Supplementary Information). The VAP zone tailing was included in the peak area integration as explained. The coefficient of determination R^2^ was 0.9874, which was still acceptable given the manual application. As a result, 5 ng/band VAP was calculated for the quantitatively analyzed refined palm kernel oil (Fig. 3b), which was near the expected fortified 6 ng/band VAP.

In addition, the manual performance of the experiment was repeated for sunflower seed oil (Fig. 3c), which showed a coefficient of determination R^2^ of 0.9999 which was exactly as good as the result generated via the application using professional commercial equipment. The calculated amount of VAP was 5 ng/band, while 5 ng/band was expected. This showed that a very good accuracy can be reached despite a simple method performance as well as a still acceptable accuracy for challenging oil matrices as given for refined palm kernel oil. Thus, the potential of the HPTLC technique was successfully demonstrated with sunflower seed oil containing a relatively simple matrix and refined palm kernel oil containing a relatively more complex matrix.

### Newly proposed RP–HPTLC–FLD test kit for VAP analysis

**Fig. 4:**
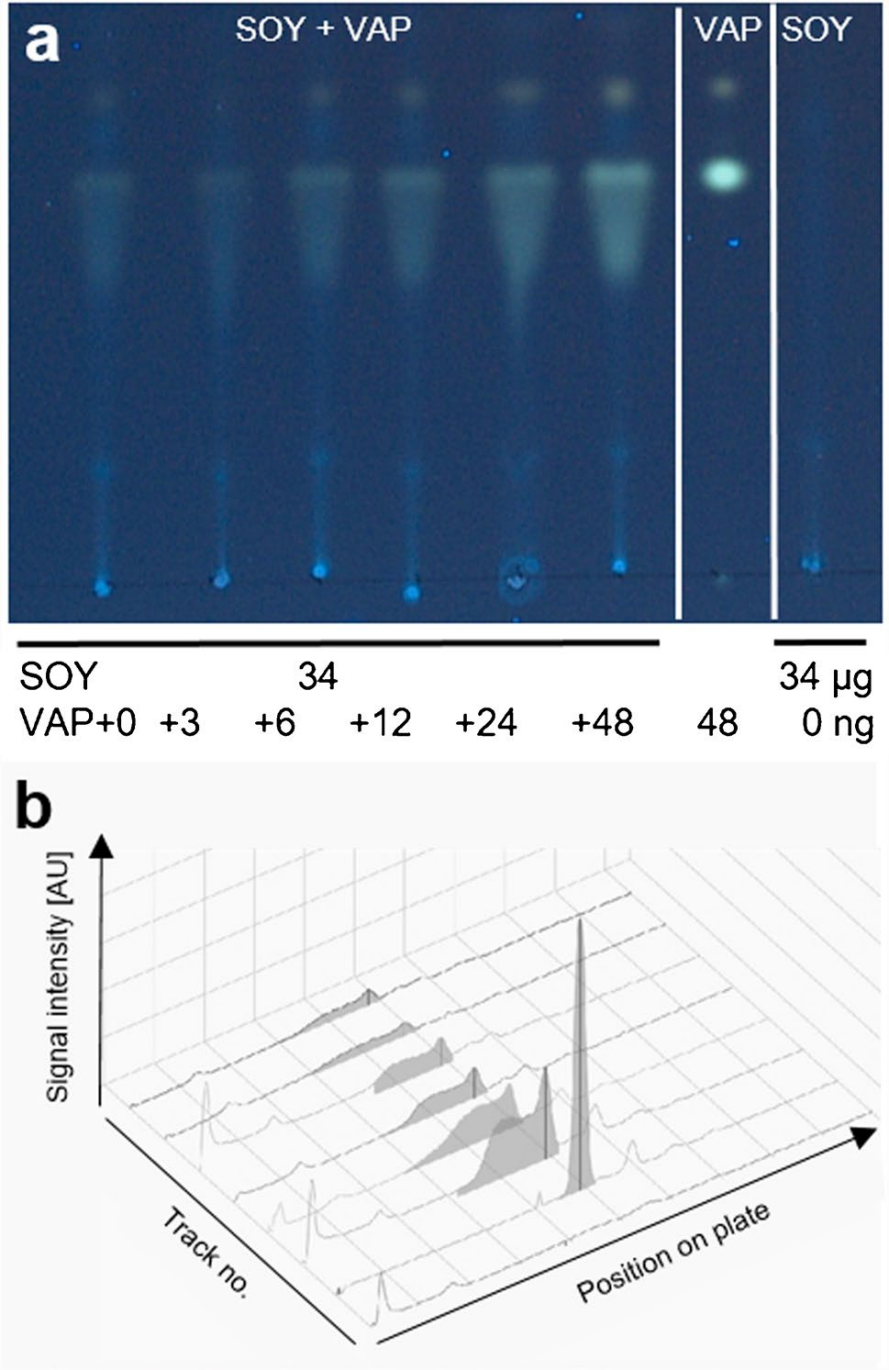
Manual performance of the method and soybean oil analysis: **a** HPTLC chromatogram at FLD 366 nm on HPTLC plate RP-18 developed with acetone–water 18:1 (*V*/*V*) and **b** respective videodensitogram of diluted soybean oil (SOY; 34 µg/band each) spiked with increasing amounts of vitamin A palmitate (VAP, 3–48 ng/band) in comparison to the references VAP (48 ng/band) and SOY (34 µg/band)

Important progress was achieved in contrast to the status quo. Key improvements were reached by the chromatographic separation (of VAP from matrix interferences), the sensitive detection (VAP fluorescence enhancement in the apolar matrix), and the calibration in the matrix (good method trueness via the standard addition method) providing accurate results even for difficult-to-analyze oil samples for the first time. Thus, the developed RP–HPTLC–FLD method provided selective, sensitive, and reliable detection of VAP and was found to be a promising tool for a simple quantitative analysis of the VAP content of oil. Using a mobile phase of acetone and water (18:1, *V*/*V*) instead of ethanol reduces the time for plate development to less than 15 min. The HPTLC chromatogram at FLD 366 nm and the respective videodensitogram of the diluted soybean oil clearly showed the separation of VAP from any interfering matrix (Fig. 4). It demonstrated that the faster mobile phase was appropriate for the analysis, which was confirmed for the proposed test kit.

**Fig. 5:**
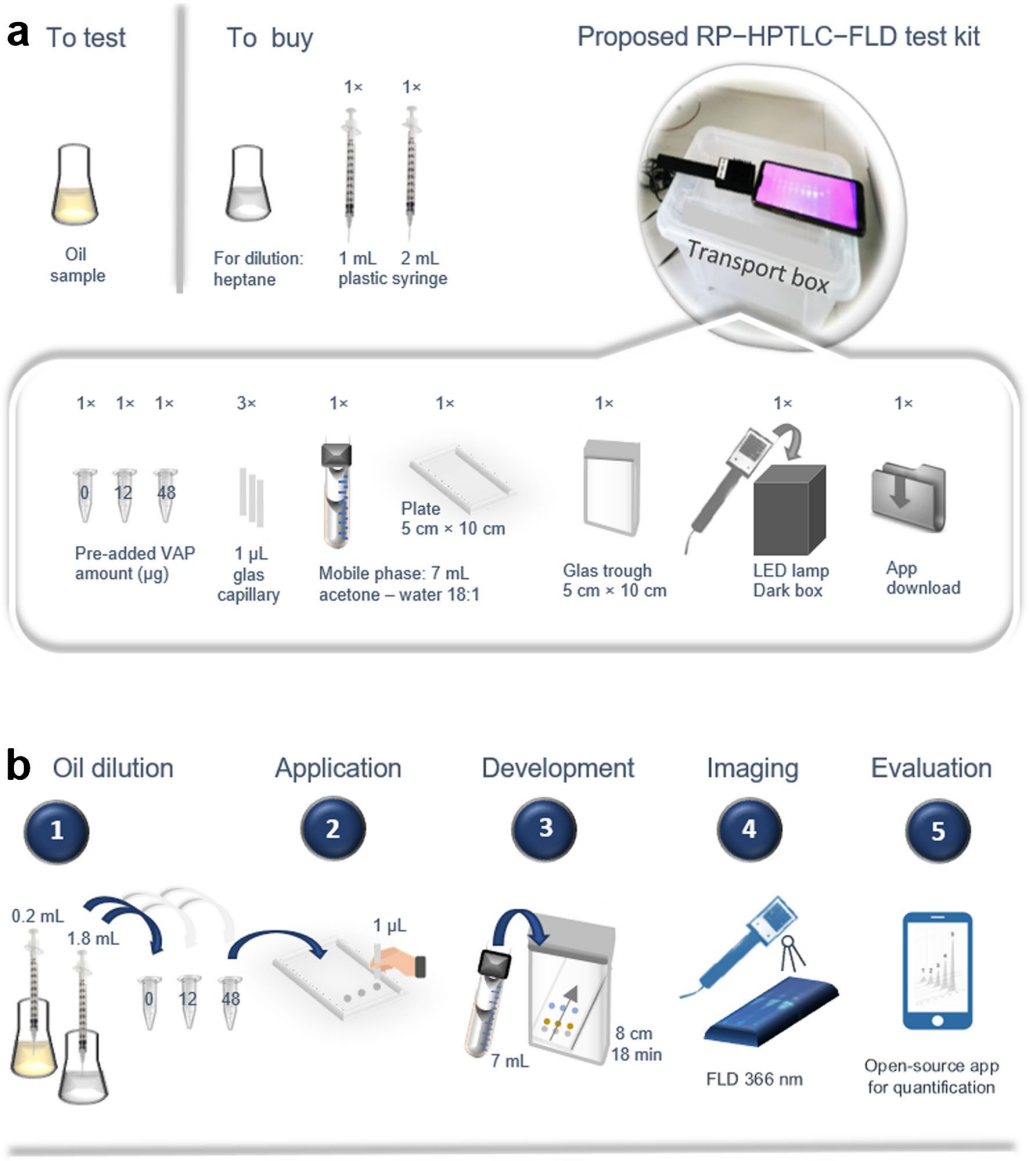
Proposed RP–HPTLC–FLD test kit for VAP analysis: **a** required items being sustainable, low hazard, rapid, and simple in performance and operation, requiring per oil sample only small volumes of *n-*heptane for oil dilution, and acetone and water for separation (together < 5 mL) and **b** scheme of the manual workflow steps (no. 1–5)

Given all these improvements, a new RP–HPTLC–FLD test kit was designed and proposed (Fig. 5a). Since large and expensive HPTLC instruments including sophisticated software are not needed, the potential for low-cost, sustainable, and portable analysis is evident. The operation is based on manual sample application, separation by capillary forces, simple image recording of sensitive and selective fluorescence signals, and electronic evaluation via app and smartphone devices and potential autonomous videodensitometry. The sustainability is evident by consuming altogether less than 5 mL of solvents per oil sample of low-level toxicity chemicals (*n*-heptane, acetone, and water) and requiring power supply only for the image evaluation part.

As workflow steps (Fig. 5b), a vial could be filled with a given volume of oil and then filled up with *n*-heptane to obtain the required 1:10 dilution. This would be repeated twice for vials containing two different VAP amounts (VAP pre-added in both vials) needed for the standard addition method (3-point calibration). To apply sharp spots and reduce the zone tailing observed for manual application, a calibrated 1-µL glass capillary is recommended for untrained users using respectively adjusted concentrations. Experts do quantification via calibrated 1-µL or 2-µL capillaries almost as accurately as via automated devices, but the 1-µL capillary ensures a sharp start zone for untrained users following a by-packed image-based instruction for its usage. After application of each sample onto an HPTLC plate and fast development via acetone–water 18:1 (about 15 min), the chromatogram would be documented via the smartphone in a dark box with illumination at 366 nm using respective low-cost LEDs and a Raspberry PI camera as described [28–32]. Any software capable of generating signal profiles [33] on a smartphone [34] can be used to autonomously generate the calibration curve, which then can be used for automatic calculation and presentation of the VAP content of the oil.

The proposed RP–HPTLC–FLD test kit for VAP analysis is considered a good scientific basis and inspiration to develop a commercial one in the near future. Another low-cost and open-source instrumental alternative is to use the sustainable, portable, miniaturized, all-in-one 2LabsToGo-Eco system [28–32]. It can be set up at the production site and is very useful when different HPTLC analyses have to be performed. It is automated and can be used for automated application, development, and documentation of the oil samples with the developed methodology. Open-source software can be used for quantification [33] and further evaluation [35, 36] of the recorded chromatogram image.

## Conclusions and outlook

The developed RP–HPTLC–FLD method for quantification of VAP was demonstrated to function for five different plant oils, including those with the most difficult oil matrices. The method uses only low-hazard reagents, which are *n-*heptane (for oil dilution) and a mixture of acetone and water (for separation). It is thus more sustainable and acceptable for the environment compared to other methods and suited for analysis even outside the laboratory setting. The new RP–HPTLC–FLD method showed acceptable coefficients of determination and accuracies evaluated via the standard addition method. Thus, it allowed for the quantification of VAP in the tested edible plant oils. The proposed RP–HPTLC–FLD test kit for VAP analysis is easy to use with low-tech equipment. It is intended for quantitative and affordable cost-efficient on-site analysis in oil mills to meet the demand from small- and medium-sized enterprises and various technical support organizations active in developing countries. It supports transparency and security in the oil fortification value chain, which is essential for millions of people who rely on sufficient daily intake of vitamin A.

## Supplementary Information

Below is the link to the electronic supplementary material. 

ESM1 (PDF 756 KB)

## Data Availability

The authors integrated additional data in the Supplementary Information and further information is made available on request.

## References

[1] Zhao T, Liu S, Zhang R, Zhao Z, Yu H, Pu L, *et al*. Global Burden of Vitamin A Deficiency in 204 Countries and Territories from 1990–2019. Nutrients. 2022;14(5). 10.3390/nu14050950 PMC8912822 35267925

[2] Stevens GA, Beal T, Mbuya MNN, Luo H, Neufeld LM. Global Micronutrient Deficiencies Research G. Micronutrient deficiencies among preschool-aged children and women of reproductive age worldwide: a pooled analysis of individual-level data from population-representative surveys. Lancet Glob Health. 2022;10(11):e1590-e9. 10.1016/S2214-109X(22)00367-9 PMC10918648 36240826

[3] Yue T, Zhang Q, Li G, Qin H. Global Burden of Nutritional Deficiencies among Children under 5 Years of Age from 2010 to 2019. Nutrients. 2022;14(13). 10.3390/nu14132685 PMC9268233 35807863

[4] Keats EC, Neufeld LM, Garrett GS, Mbuya MNN, Bhutta ZA. Improved micronutrient status and health outcomes in low- and middle-income countries following large-scale fortification: evidence from a systematic review and meta-analysis. Am J Clin Nutr. 2019;109(6):1696–708. 30997493 10.1093/ajcn/nqz023 PMC6537942

[5] Blüthner A, Vierck L. Setting Standards for Business and Development – How legal Frameworks can support market-based Nutrition Partnerships. European Food and Feed Law Review. Berlin, Germany: Lexxion; 2009.

[6] Fiedler JL, Lividini K, Bermudez OI. Estimating the impact of vitamin A-fortified vegetable oil in Bangladesh in the absence of dietary assessment data. Public Health Nutr. 2015;18(3):414–20. 24762782 10.1017/S1368980014000640 PMC10271769

[7] Raghavan R, Aaron GJ, Nahar B, Knowles J, Neufeld LM, Rahman S, et al. Household coverage of vitamin A fortification of edible oil in Bangladesh. PLoS ONE. 2019;14(4): e0212257. 30943194 10.1371/journal.pone.0212257 PMC6447147

[8] Walters D, Ndau E, Saleh N, Mosha T, Horton S. Cost-effectiveness of sunflower oil fortification with vitamin A in Tanzania by scale. Matern Child Nutr. 2019;15(Suppl 3): e12720. 31148403 10.1111/mcn.12720 PMC6593718

[9] Osendarp SJM, Martinez H, Garrett GS, Neufeld LM, De-Regil LM, Vossenaar M, et al. Large-Scale Food Fortification and Biofortification in Low- and Middle-Income Countries: A Review of Programs, Trends, Challenges, and Evidence Gaps. Food Nutr Bull. 2018;39(2):315–31. 29793357 10.1177/0379572118774229 PMC7473077

[10] Dary O, Mora JO. Food fortification to reduce vitamin A deficiency: International Vitamin A Consultative Group recommendations. J NUTR. 2002;132(9 Suppl):2927S-2933S. 12221271 10.1093/jn/132.9.2927S

[11] Allen L, de Benoist B, Dary O, Hurreell R. Guidelines on Food Fortification with Micronutrients. Geneva: World Health Organization; 2006.

[12] Jungjohann SM, Ara G, Pedro C, Friesen VM, Khanam M, Ahmed T, *et al*. Vitamin A Fortification Quality Is High for Packaged and Branded Edible Oil but Low for Oil Sold in Unbranded, Loose Form: Findings from a Market Assessment in Bangladesh. Nutrients. 2021;13(3). 10.3390/nu13030794 PMC7997297 33670884

[13] Rimkus GG, Schubert M, Morgan D, Jungjohann S. Rapid direct analysis of retinyl palmitate (vitamin A) in fortified vegetable oils by HPLC-FLD. Food Addit Contam Part A Chem Anal Control Expo Risk Assess. 2022;39(1):24–34. 34587464 10.1080/19440049.2021.1977854

[14] Kumar A, Kamboj M, Virender. A review on photometric methods for the quantitation of vitamin A. Microchem. J. 2021;171.

[15] Renaud C, Berger J, Laillou A, Avallone S. Quantification of vitamin A in fortified rapeseed, groundnut and soya oils using a simple portable device: comparison to high performance liquid chromatography. Int J Vitam Nutr Res. 2013;83(2):122–8. 24491885 10.1024/0300-9831/a000154

[16] Huey SL, Krisher JT, Morgan D, Mkambula P, Srinivasan B, Gannon BM, et al. Portable Devices for Measurement of Vitamin A Concentrations in Edible Oil: Field Readiness of Available Options. ACS Omega. 2022;7(21):17502–18. 35664625 10.1021/acsomega.1c07181 PMC9161250

[17] Palma-Duran SA, Morgan D, Combet E. Quantification of Vitamin A in Edible Oils: Comparison of Portable Device iCheck Chroma3 to High-Performance Liquid Chromatographhy. Food Anal Methods. 2024;17:847–54. 38765762 10.1007/s12161-024-02613-w PMC11101343

[18] Carr FH, Price EA. Colour reactions attributed to vitamin A. Biochem J. 1926;20:497–501. 16743683 10.1042/bj0200497 PMC1251741

[19] Kildahl-Andersen G, Naess SN, Aslaksen PB, Anthonsen T, Liaaen-Jensen S. Studies on the mechanism of the Carr-Price blue colour reaction. Org Biomol Chem. 2007;5(18):3027–33. 17728870 10.1039/b709535j

[20] The_United_States_Pharmacopeial_Convention. Vitamin A Oral Liquid Preparation. The United States pharmacopeia (USP) The National formulary: Rockville, Md. : United States Pharmacopeial Convention, Inc.; 2022.

[21] European_Directorate_for_the_Quality_of_Medicines_&_HealthCare_(EDQM). Vitamin A concentrate (oily form), synthetic European Pharmacopoeia (Ph Eur). 10th Edition ed: EUR-Lex; 2021.

[22] Cimpoiu C, Hosu A. Thin Layer Chromatography for the Analysis of Vitamins and Their Derivatives. J Liq Chromatogr Relat Technol. 2007;30:28.

[23] HPTLC Identification of Fatty Oils (Fixed Oils), Application Notes. CAMAG Laboratory 2014. https://chemexcil.in/uploads/files/F-39_HPTLC_Identification_of_Fatty_Oils_Fixed_Oils_USP.pdf. Accessed 31 January 2025.

[24] Jadoon A, Malik A, Qazi MH, Azis M. Spectrophotometric method for the determination of Vitamin A and E using Ferrozine-Fe(II) complex. Asian J. Chem. 2013;6(4).

[25] Stahl E, Schild W. Isolierung und Charakterisierung von Naturstoffen 1. Stuttgart/New York: Auflage. Gustav Fischer Verlag GmbH; 1986.

[26] Matissek R, Steiner G, Fischer M. Lebensmittelanalytik. 4th ed. Berlin: Springer; 2010.

[27] Bryl K, Drabent R, Olszewska T. Fluorescent Properties of Vitamin a Derivative, Retinyl Palmitate, in Binary Solvents with Aqueous Phase. Spectrosc Lett. 1998;31:647–58.

[28] Schade F, Schwack W, Demirbas, Y, Morlock, G.E. Open-source all-in-one LabToGo Office Chromatography. Anal. Chim. Acta 2021;1174:338702. 10.1016/j.aca.2021.338702 34247737

[29] Sing L, Schwack W, Göttsche R, Morlock GE. 2LabsToGo − Recipe for building your own chromatography equipment including biological assay and effect-detection. Anal Chem. 2022;94:14554–64. 36225170 10.1021/acs.analchem.2c02339 PMC9610689

[30] Morlock GE, Koch J, Schwack W. Miniaturized open-source 2LabsToGo screening of lactose-free dairy products and saccharide-containing food. J Chromatogr A. 2023;1688: 463720. 36566572 10.1016/j.chroma.2022.463720

[31] Jakob K, Schwack W, Morlock GE. All-in-one 2LabsToGo system for analysis of ergot alkaloids in whole rye. Food Chem. 2024;453: 139593. 38761724 10.1016/j.foodchem.2024.139593

[32] Romero MCO, Jakob K, Schmidt J, Nimmerfroh T, Schwack W, Morlock GE. Consolidating two laboratories into the most sustainable lab of the future: 2LabsToGo-Eco, Anal Chim Acta. 2025. 10.1016/j.aca.2025.344103. 10.1016/j.aca.2025.344103 40610132

[33] Fichou D, Morlock GE. quanTLC, an online open-source solution for videodensitometric quantification. J Chromatogr A. 2018;1560:78–81. 29789168 10.1016/j.chroma.2018.05.027

[34] Hauk C, Boss M, Gabel J, Schäfermann S, Lensch HPA, Heide L. An open-source smartphone app for the quantitative evaluation of thin-layer chromatographic analyses in medicine quality screening. Sci Rep. 2022;12:13433. 35927306 10.1038/s41598-022-17527-y PMC9352711

[35] Fichou D, Morlock GE. Powerful artificial neural network for planar chromatographic image evaluation, shown for denoising and feature extraction. Anal Chem. 2018;90:6984–91. 29708729 10.1021/acs.analchem.8b01298

[36] Fichou D, Ristivojevic P, Morlock GE. Proof-of-principle of rTLC, an open-source software developed for image evaluation and multivariate analysis of planar chromatograms. Anal Chem. 2016;88:12494–501. 28193066 10.1021/acs.analchem.6b04017

