# Supplementary material for: Proposed RP–HPTLC–FLD kit for analysis of vitamin A palmitate in edible oils
Source: Anal Bioanal Chem. 2025 May 19;417(17):3791–801. doi: 10.1007/s00216-025-05894-0 (PMC12227463; doi:10.1007/s00216-025-05894-0)
Supplement: Supplementary file 1 — (PDF 756 KB) [file 216_2025_5894_MOESM1_ESM.pdf]

Supplementary Material

Proposed RP−HPTLC−FLD kit for analysis of vitamin A palmitate in edible oils

Daniel Meyer[1], Sophia Hörnlein[1], Dietrich Rein[2], Gertrud E. Morlock[1,*]

[1]Institute of Nutritional Science, Chair of Food Science, Justus Liebig University Giessen, Heinrich-Buff-Ring 26-32, 35392 Giessen, Germany

[2]BASF SE, Carl-Bosch-Str. 38, 67056 Ludwigshafen, Germany

*Corresponding author. Tel.: +49 641 9939141; fax: +49 641 9939149. E-mail address: Gertrud.Morlock@uni-giessen.de (G.E. Morlock).

GEM: https://orcid.org/0000-0001-9406-0351

DM: https://orcid.org/0000-0002-1505-9529

DR: https://orcid.org/0000-0003-0076-1865

Table S1 Procedure data for spiking of oils with vitamin A palmitate (VAP)

Oils used for	VAP			Weight 200 μL oil	Calculated VAP	Added VAP	Applied VAP	Volume [μL] of VAP (10 ng/μL) for overspray					
**Overspray**	[IU/kg]	[μg/kg]	[μg/mg]	[mL]	[μg]	[μL] (1 mg/mL) to 200 μL oil	per diluted oil [ng/2 μL]	**Band 1 (+0)**	**Band 2 (+3)**	**Band 3 (+6)**	**Band 4 (+12)**	**Band 5 (+24)**	**Band 6 (+48)**
Sunflower	25000	13750	0.01375	154	2.1	2.1	2.1	2	0.3	0.6	1.2	2.4	4.8
Linseed	25000	13750	0.01375	170	2.3	2.3	2.3	2	0.3	0.6	1.2	2.4	4.8
									**Volume [μL] of VAP (100 ng/μL) + 0.2 mL fortified oil**		**Volume [μL] of VAP (1000 ng/μL) + 0.2 mL fortified oil**		
**Fortification**								**Band 1 (+0)**	**Band 2 (+3)**	**Band 3 (+6)**	**Band 4 (+12)**	**Band 5 (+24)**	**Band 6 (+48)**
Unrefined palm	65000	35750	0.01375	184	6.6	6.6	6.6	7	3	6	1.2	2.4	4.8
Refined palm	65000	35750	0.01375	166	5.9	5.9	5.9	6	3	6	1.2	2.4	4.8
Sunflower	65000	35750	0.01375	160	5.7	5.7	5.7	6	3	6	1.2	2.4	4.8
Soybean	65000	35750	0.01375	171	6.1	6.1	6.1	6	3	6	1.2	2.4	4.8

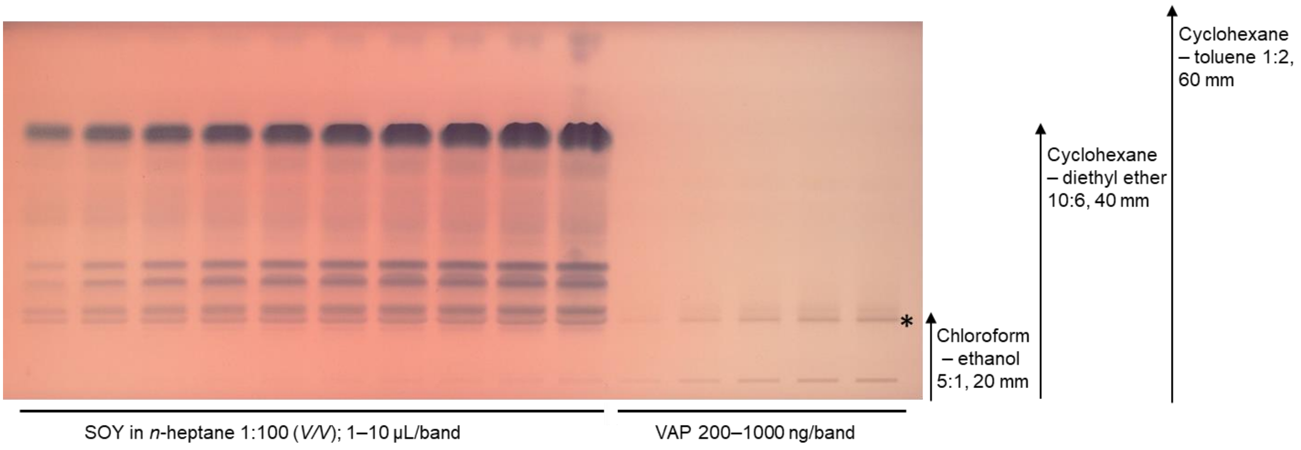

Fig. S1 HPTLC–Vis chromatogram at white light illumination after derivatization via the 4-methoxybenzaldehyde reagent of soybean oil (SOY) in comparison to the vitamin A palmitate (VAP: marked*), both applied in increasing amounts on the HPTLC plate RP-18 and separated via an ascending 3-step development as indicated.

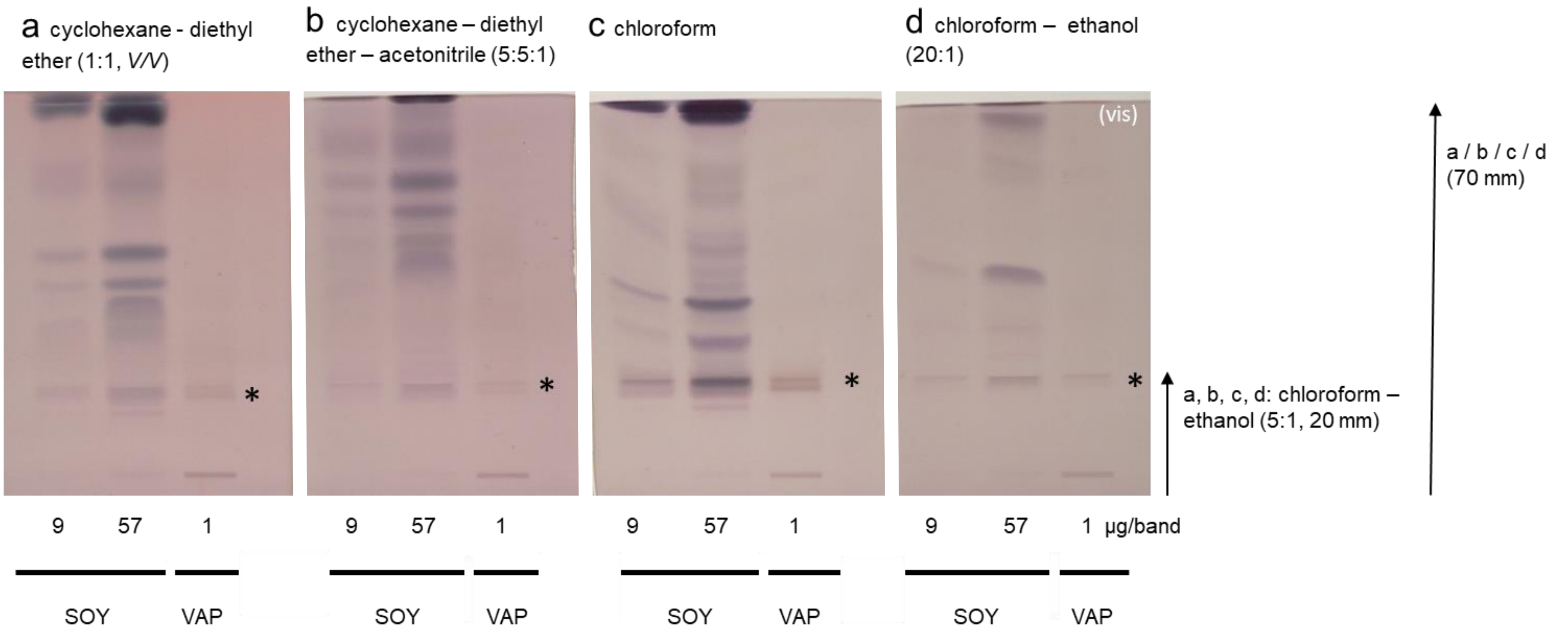

Fig. S2 HPTLC–Vis chromatograms (a-d) at white light illumination after derivatization with the 4-methoxybenzaldehyde reagent of diluted soybean oil (SOY, 9 and 57 µg/band, 1 and 7 µL/band of a 1:100 dilution in isopropanol, *V/V*) in comparison to the vitamin A palmitate (VAP: marked*;1 µg/band, 10 µL/band of a 100-ng/µL solution) separated on normal phase HPTLC plate silica gel 60 with the indicated mobile phase system. For derivatization, the plate was immersed (2 cm/s, 2 s) in the derivatization reagent (1.5 g 4-methoxybenzaldehyd dissolved in 210 mL methanol + 25 mL acetic acid + 13 mL concentrated sulfuric acid) and heated at 120°C for 10 min.

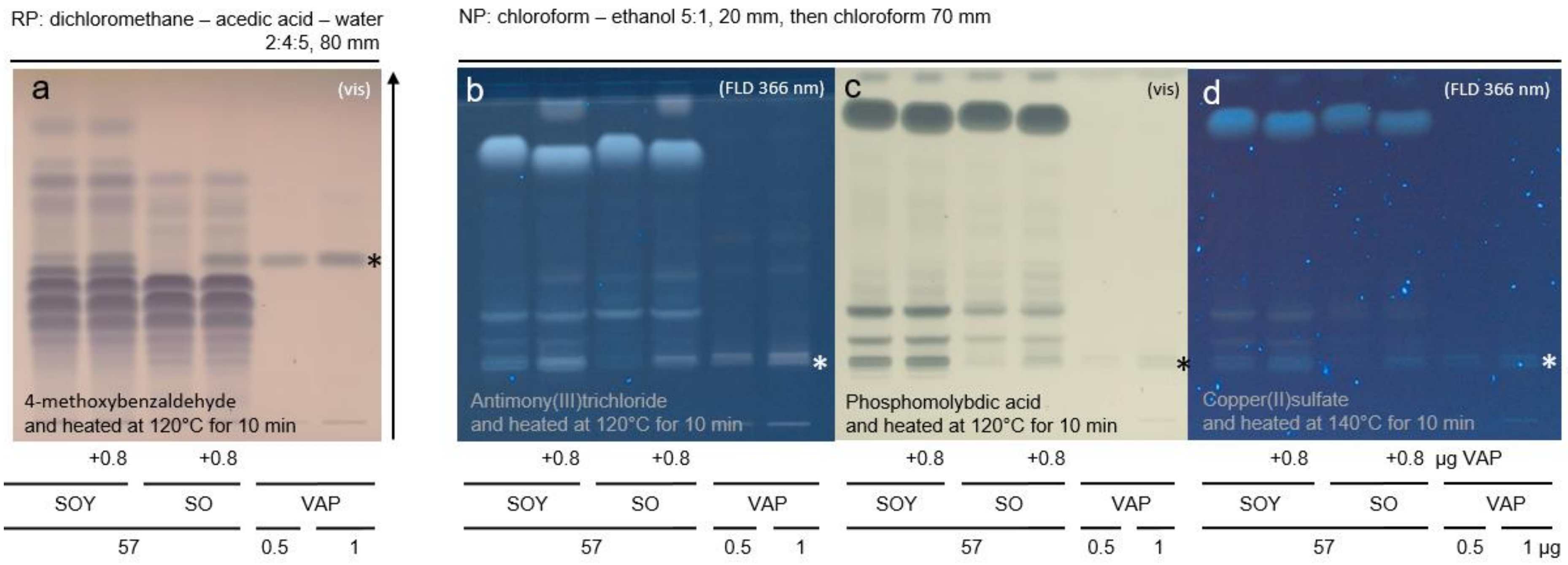

Fig. S3 RP-/NP-HPTLC chromatograms (a-d) at white light illumination after derivatization with different derivatization reagents (a/c) and at FLD 366 nm (b/d) of diluted soybean oil (SOY, 57 µg/band, 7 µL/band of a 1:100 dilution in isopropanol, *V/V*), the same mixed 1:1 (*V/V*) with vitamin A palmitate (VAP) standard solution (100 ng/µL) yielding a 50 VAP ng/µL solution (57 µg SOY/band together with 0.8 µg VAP/band, 14 µL/band), diluted sunflower seed oil (SO, 57 µg/band, 7 µL/band of a 1:100 (*V/V*) in isopropanol), the same mixed with the same VAP solution yielding the same solution but with SO (57 µg SO/band together with 0.8 µg VAP/band) and VAP (0.5 and 1 µg/band), separated on reversed phase (RP) HPTLC plate RP-18 and normal phase (NP) HPTLC plates silica gel 60. For derivatization, the plate was immersed (2 cm/s, 2 s) in:

- 4-Methoxybenzaldehyd reagent (1.5 g 4-methoxybenzaldehyd dissolved in 210 mL methanol + 25 mL acetic acid + 13 mL concentrated sulfuric acid) and heated at 120°C for 10 min.
- Antimony(III)trichloride reagent (2 g antimony(III)trichloride dissolved in 50 mL chloroform) and heated at 120°C for 10 min.
- Phosphomolybdic acid reagent (20 g phosphomolybdic acid hydrate dissolved in 100 mL ethanol) and heated 120°C for 10 min.
- Copper(II)sulfate reagent (1.6 g copper(II)sulfate + 40 mL water) and heated at 120°C for 10 min.

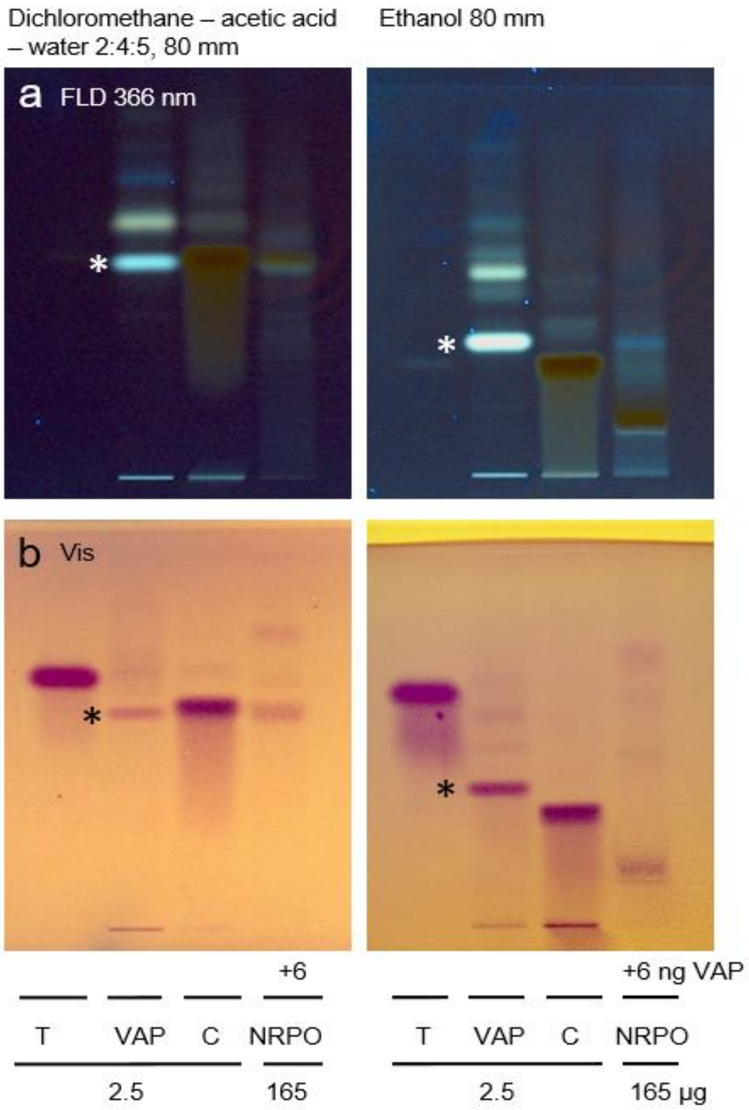

Fig. S4 Derivatization with the iron (III) chloride/ferrozine reagent: (a) HPTLC RP-18 chromatograms at FLD 366 nm and (b) white light illumination after derivatization with the iron (III) chloride/ferrozine reagent showing α-tocopherol (T, 2.5 µg/band), vitamin A palmitate (VAP, 2.5 µg/band, marked*), β-carotene (C, 2.5 µg/band) and diluted not refined palm oil (NRPO, 165 µg/band) spiked with VAP (6 ng/band) separated on HPTLC plates RP-18 with two different mobile phase systems as indicated. The plate was immersed (2 cm/s, 1 s) first in the iron (III) chloride solution (8 mg in 100 mL ethanol), then ferrozine solution (50 mg in 2.5 mL ethanol and 0.5 mL water) was sprayed onto the plate and heated at 120 °C for 10 min.

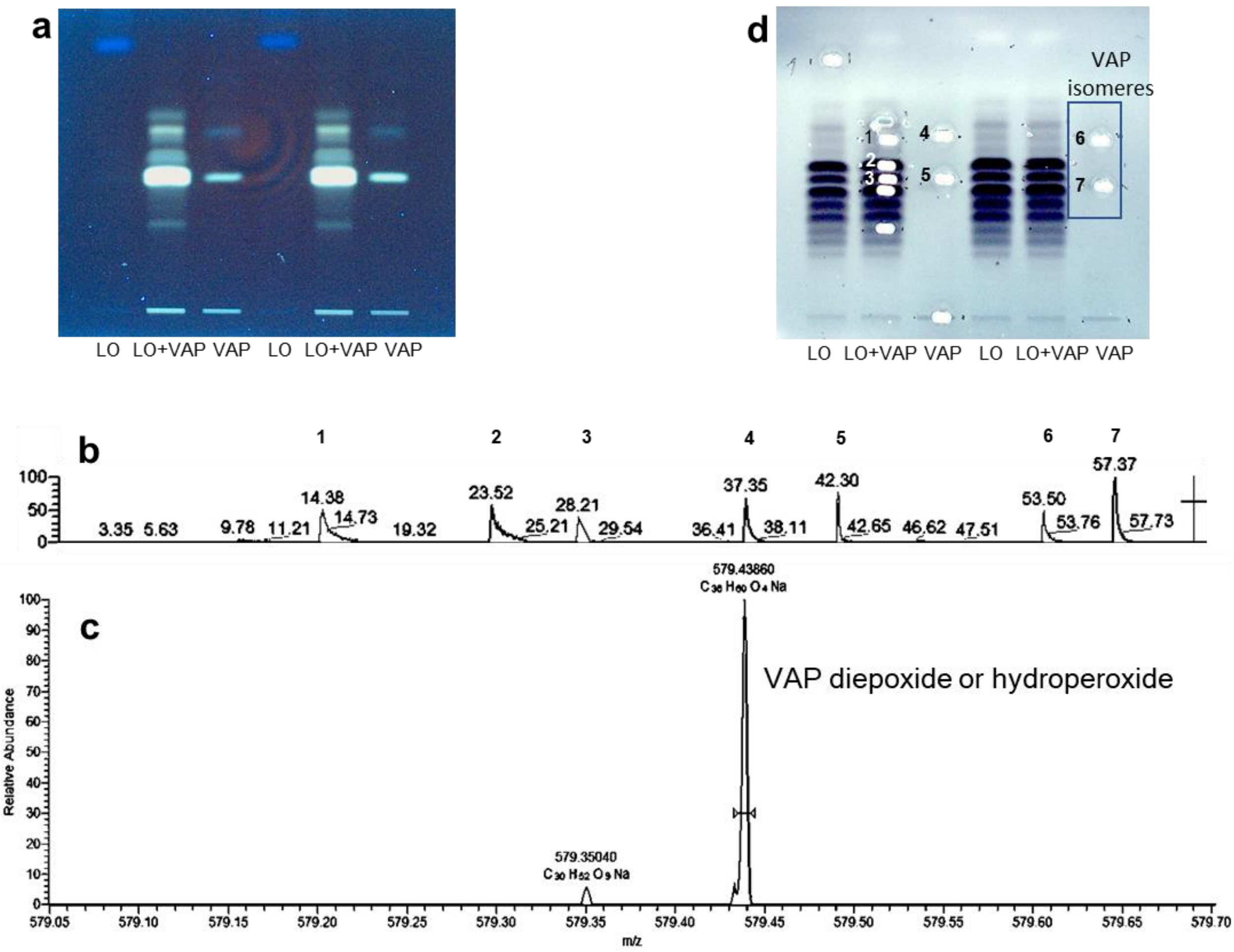

Fig. S5 Study of selected zones evident in the vitamin A palmitate (VAP) reference track: (a) HPTLC chromatogram at FLD 366 nm of diluted linseed oil (7 µL/band of 1:100 dilution in isopropanol, *V/V*), the same containing 1.4 µg VAP (LO+VAP), and VAP (1.4 µg/band) separated on an HPTLC plate silica gel 60 RP-18 with dichloromethane – acetic acid – water 2:4:5 (*V/V/V)* up to 80 mm; (b) selected ion chromatogram of high-resolution mass spectrometry recording of the eluted zones (at the times marked 1–7, which correspond to the eluted zones 1–7 (d; marked) showing only one VAP sodium adduct and (c) its respective mass signal in the positive ionization mode which is the same for all eluted zones 1–7 (d; marked); (d) after zone elution, zones on the same plate were detected at while light illumination via derivatization using the phosphomolybdic acid reagent for confirmation of the proper positioning of the elution head on the zone and its elution.
